# The Association of Thyroid Disease with Risk of Dementia and Cognitive Impairment: A Systematic Review

**DOI:** 10.3390/medicina60121917

**Published:** 2024-11-21

**Authors:** Silvija Valdonė Alšauskė, Ida Liseckienė, Rasa Verkauskienė

**Affiliations:** 1 Family Medicine Clinic, Lithuanian University of Health Sciences, 50140 Kaunas, Lithuania; 2 Faculty of Medicine, Medical Academy, Lithuanian University of Health Sciences, 44307 Kaunas, Lithuania; 3 Endocrinology Clinic, Lithuanian University of Health Sciences, 50140 Kaunas, Lithuania

**Keywords:** thyroiditis, thyroid disease, hypothyroidism, hyperthyroidism, cognitive decline, cognitive dysfunction, cognitive impairment, dementia

## Abstract

*Background and Objectives*: Cognitive impairment is defined as a reduced ability to perform one or more cognitive functions, potentially leading to dementia if the condition worsens. With dementia being a rapidly growing public health issue affecting approximately 50 million people worldwide, understanding modifiable risk factors such as thyroid disease is crucial for prevention and early diagnosis. Thyroid hormones play a vital role in brain development and functioning, impacting processes such as neuron growth, myelination, and neurotransmitter synthesis. Recent decades have seen thyroid disorders emerging as potential independent risk factors for reversible cognitive impairment. *Materials and Methods*: The review adheres to PRISMA guidelines, utilizing a structured PICO question to explore whether individuals with thyroid diseases have a higher risk of developing dementia and cognitive impairments compared to those without. The literature search was conducted in PubMed, Cochrane, and ScienceDirect databases, including studies published from 1 January 2019 to 31 December 2023. The literature review discusses nine selected articles. *Results*: The findings highlight a complex association between thyroid dysfunction and cognitive decline, with some studies indicating significant links, particularly with hypothyroidism, and others suggesting the relationship may depend on the specific type of thyroid dysfunction or cognitive domain affected. Six out of nine articles found a link between thyroid disease and cognitive impairment, while three articles refuted this link. *Conclusions*: The review reveals a complex and ambiguous relationship between thyroid dysfunction and cognitive impairment. Further research is needed to elucidate the mechanisms underlying these associations and to determine whether thyroid dysfunction may be a modifiable risk factor for dementia.

## 1. Introduction

Cognitive impairment refers to the reduced ability to perform cognitive functions, impacting everyday activities. When severe, it is termed dementia, a major public health issue affecting 50 million people globally, with 10 million new cases annually, expected to triple by 2050 [1,2]. Dementia is the 7th leading cause of death and a significant cause of disability in older adults [3]. Studies estimate that thyroid dysfunction may contribute up to 10% of reversible cognitive impairment cases [4]. With no effective treatments available, prevention and early diagnosis are vital, emphasizing the importance of addressing modifiable risk factors. Dementia is a diverse group of neurodegenerative disorders, each with distinct pathogenesis and clinical signs. The primary types include Alzheimer’s disease, characterized by amyloid plaque deposition and tau neurofibrillary tangles, and vascular dementia, often resulting from reduced blood flow and microvascular damage in the brain. Depression, frequently comorbid with dementia, involves neurochemical imbalances—most notably of serotonin and norepinephrine—and inflammatory responses that can impact brain health and cognitive function [3].

This complex interplay between mood disorders and cognitive health highlights the importance of thyroid function, as thyroid hormones are integral to brain development, influencing neurogenesis, neurotransmitter regulation, and synaptic plasticity. Thyroid disorders, especially in aging populations, may thus act as modifiable risk factors for both mood and cognitive disorders, underscoring the potential for thyroid health monitoring in dementia prevention [5]. Thyroid hormones have a variety of target genes, many of which are essential for brain function and more than 1100 of which are important for brain development [6]. They regulate key genes such as the amyloid precursor protein (APP) and brain-derived neurotrophic factor (BDNF) [7,8]. Over the last two decades, thyroid diseases have been recognized as potential independent risk factors for reversible cognitive impairment [9]. Hyperthyroidism often leads to anxiety and mood disorders, while hypothyroidism is linked to cognitive impairment, particularly in the elderly [4]. Age-related changes in thyroid function complicate assessment, with altered hormone production and metabolism [10]. In healthy people between 61 and 90 years of age, secretion of both thyroxine (T4) and T3 is reduced, but serum levels of hormones, especially T4, may be normal or even slightly elevated. This is due to the slower breakdown of T4 in the older body [10]. Some studies have questioned the link between ageing and progressive thyroid dysfunction. The reduced thyroid-stimulating hormone (TSH) and higher free thyroxine (fT4) levels observed in older age are explained by reduced clearance of thyroid hormones in the liver, which is thought to inhibit the secretion of TSH, resulting in lower serum hormone levels [11]. However, several cross-sectional studies have found a high prevalence of subclinical thyroid disease among patients with dementia, when fT4 levels were normal and TSH higher or lower [12,13]. When considering subtle hormonal changes, subclinical thyroid dysfunction is worth mentioning. Subclinical thyroid dysfunction, though often mild, can influence multiple systems in the body, including the cardiovascular system. Recent studies have shown that even minor fluctuations in thyroid hormone levels can correlate with markers of subclinical cardiovascular dysfunction, such as the ankle-brachial index (ABI) [14]. This connection is particularly relevant in the context of dementia, as cardiovascular risk factors like atherosclerosis and impaired vascular health contribute significantly to the development of vascular dementia. The subtle cardiovascular impacts of subclinical thyroid dysfunction could, therefore, exacerbate neurovascular issues, creating a potential pathway for cognitive decline in individuals with underlying thyroid irregularities. This makes thyroid function screening tests important, especially when there is cognitive impairment, as treating thyroid dysfunction might improve cognitive outcomes. This systematic review aims to investigate the association between thyroid disease and cognitive impairment by evaluating publications from the last 5 years.

## 2. Materials and Methods

This systematic literature review follows the Preferred Reporting Items for Systematic Reviews and Meta-Analyses (PRISMA) guidelines [15]. Based on these guidelines and in order to structure the main question, the question of this review was formulated using the PICO principle: P (Participants)—people with thyroid disease; I (Intervention)—presence of thyroid disease; C (Comparison)—people without thyroid disease; O (Outcomes)—risk or incidence of dementia and cognitive impairment—whether people with thyroid disease have a higher risk or higher incidence of dementia and cognitive impairment than people without thyroid disease. The protocol for this systematic literature review has been registered with the International Register of Planned Systematic Reviews (PROSPERO; registration number CRD42024530779). The search was performed in the PubMed, Cochrane, and ScienceDirect databases between 30 January 2024 and 1 March 2024. The search included studies published in the last 5 years (1 January 2019–31 December 2023). Only studies published in English or Lithuanian were included in the analysis. Translation of the data was performed using ChatGPT version 4o, an AI language model by OpenAI. The tool was used to translate Lithuanian content into English. The translations were reviewed and validated by the authors for accuracy. Databases were searched for articles using the following keywords: “thyroiditis”, “thyroid disease”, “hypothyroidism”, “hyperthyroidism”, “cognitive decline”, “cognitive dysfunction”, “cognitive impairment”, “dementia”. The keyword combinations used are shown in Table 1.

### 2.1. Eligibility and Exclusion Criteria for Articles

Studies that met all of the following inclusion criteria were considered for inclusion: (1) they were published in English and Lithuanian, (2) a cohort, correlational, cross-sectional, or case-control study were conducted, (3) the subjects were over 18 years of age, and (4) the articles were published in the last 5 years. Studies were excluded if they had any of the following criteria: (1) only an abstract was available, (2) the article was a case study, an animal study, a letter to the editor, or a commentary.

### 2.2. Selecting and Organising Articles

The selection was carried out using inclusion and exclusion criteria via the open source web-based software Rayyan [16]. Articles were selected in stages. First, articles that matched the keywords used in the search and the activated filters (English language and human studies) were selected. Irrelevant titles were excluded. Titles and abstracts of remaining articles were reviewed, and those not meeting inclusion criteria were excluded. Final selection involved full-text review and inclusion if all criteria were met. To avoid errors in the selection of the articles, all titles were reviewed twice, and, in case of doubt, the abstracts were also reviewed again. The following data were extracted from each study and structured: study characteristics, sample characteristics, and results. The extracted and structured data elements included country, sample size, target group, thyroid diseases studied, study design, and results.

### 2.3. Assessing the Quality of Articles

The selected articles were assessed using the Newcastle–Ottawa Scale (NOS) for evaluating non-randomized studies, with criteria adapted for this review (the design of the studies differed; hence, the criteria were adapted) [17]. The assessment was divided into three parts: selection, comparability, and outcomes, each part receiving a starred rating (‘⁎’). Results of the quality assessment are presented in Table 2, with detailed items in Appendix A, Table A1.

### 2.4. Data Systematisation and Analysis

**Figure 1:**
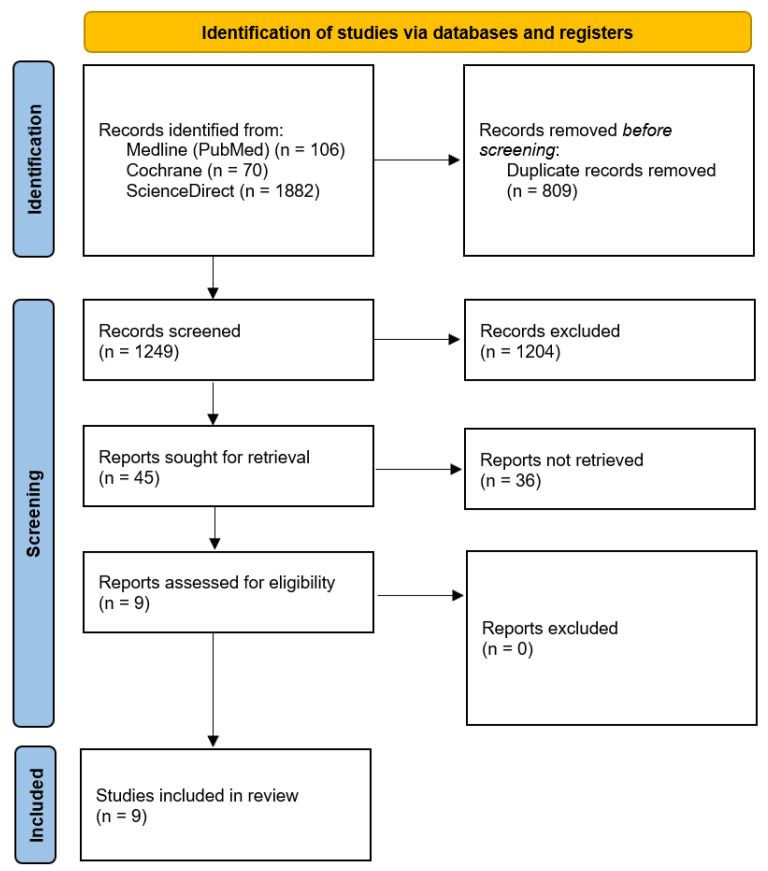
PRISMA flow diagram.

A literature review conducted on 20 February 2024 yielded 2058 articles. After removing duplicates, 1249 articles remained. Applying inclusion and exclusion criteria to titles and abstracts narrowed this to 45 articles for full-text review. Finally, 9 articles met all inclusion criteria and were included in the review. The selection process followed the PRISMA flow diagram (Figure 1) [15].

## 3. Results

The characteristics of the studies included in this literature review are presented in Table 3. The studies varied methodologically: six cohort, two case-control, and one cross-sectional design.

Conducted on four continents, five studies targeted adults without thyroid disease or cognitive impairment. Three studies focused on hypothyroidism, one specifically on women, and one on Alzheimer’s patients. All studies aimed to evaluate the relationship between thyroid disease and cognitive impairment. Six articles identified a relationship, while three refuted it.

This review considers a range of thyroid dysfunctions, including hypothyroidism, hyperthyroidism, and autoimmune thyroiditis, each with distinct cognitive implications. Notably, hypothyroidism is linked to memory deficits, while hyperthyroidism often correlates with mood and anxiety issues, complicating cognitive assessments [24].

### 3.1. Evidence Supporting the Link Between Thyroid Disease and Cognitive Impairment

Kim JH et al. identified a higher occurrence of thyroid disorders, including hypothyroidism, thyroiditis, and hyperthyroidism, among individuals with Alzheimer’s disease (AD) as shown in Table 3 [18].

The study by Sipala PN et al. revealed a complex relationship between thyroid dysfunction, particularly hypothyroidism, and an elevated risk of developing dementia. In their study, a cohort of 3416 individuals diagnosed with dementia was observed. They reported that hypothyroidism nearly doubled dementia risk over 19 years, even after adjusting for conventional risk factors [22]. This suggests that hypothyroidism itself may directly contribute to the increased risk of dementia. Additionally, data that partially corroborate the findings of this study were observed in the cohort study conducted by Thvilum et al. The results demonstrate that the relationship between hypothyroidism and the risk of developing dementia is complex and dependent on number of factors. A clear association between hypothyroidism and an elevated risk of dementia was identified in the Danish National Patient Register (DNPR) cohort. However, this association was significantly reduced after accounting for pre-existing comorbidities (such as cardiovascular diseases and diabetes), indicating that the initially observed risk of dementia may have been partially attributable to these comorbidities. Additionally, the analysis showed a complex relationship between age and dementia risk in hypothyroid subjects, with a higher risk observed in individuals younger than 56 years, while no significant risk was found in those aged 56 or older. This suggests that younger individuals with hypothyroidism may be at a disproportionately higher risk of dementia than their peers without the condition, potentially due to the more significant relative impact of TSH variations on developing neural circuits in younger adults. Furthermore, the OPENTHYRO cohort demonstrated that an elevated TSH level over an extended period was associated with an increased risk of cognitive impairment [20]. In contrast, Folkestad L. et al. concentrated on the particular effects of Graves’ disease and toxic nodular goiter on the likelihood of dementia in the OPENTHYRO registry cohort, with results that were entirely contradictory. They found that Graves’ disease and toxic nodular goiter increased AD and vascular dementia risk, with prolonged TSH reduction significantly raising all-cause dementia risk. For every six months of TSH reduction, there was a notable increase in the risk of all-cause dementia [26].

**Figure 2:**
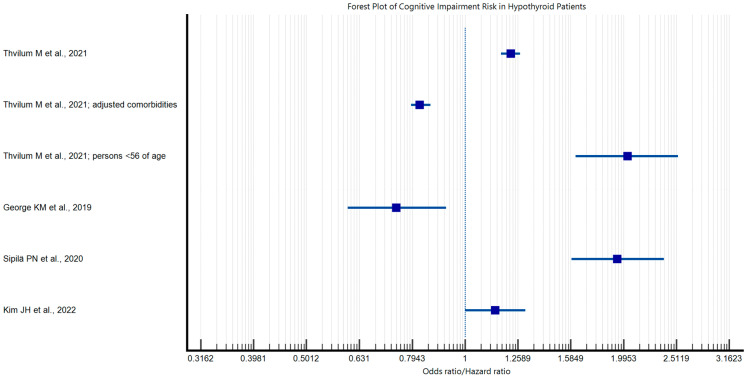
Forest plot depicting cognitive impairment risk in hypothyroidism [18,20,21,22].

**Figure 3:**
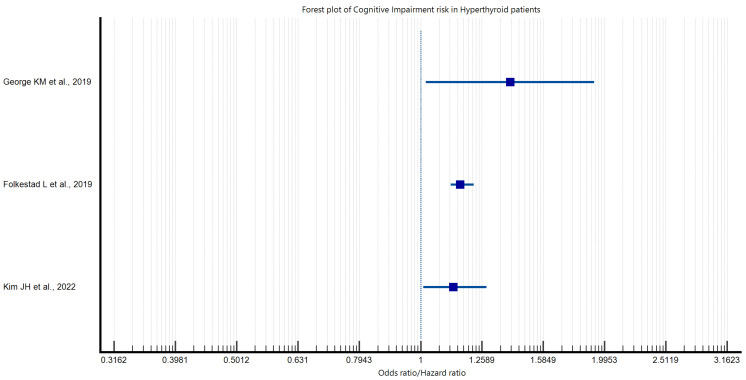
Forest plot depicting cognitive impairment risk in hyperthyroidism [18,21,26].

The relationship between female gender and thyroid disease is frequently documented in the scientific literature. Consequently, the study by Kamyshna et al. focused exclusively on women to investigate the potential correlation between thyroid disease and cognitive function. The study demonstrated that 50% of patients with post-operative hypothyroidism exhibited no cognitive impairment according to the Mini–Mental State Examination (MMSE) test for cognitive function. Conversely, 37.5% of this group demonstrated moderate cognitive impairment, while 12.5% exhibited mild dementia. Among patients with autoimmune thyroiditis (AIT) and hypothyroidism, 49.2% exhibited no cognitive impairment, 30.8% demonstrated moderate cognitive impairment, and 20% displayed mild dementia. No cases of moderate or severe dementia were observed among the patients. In the AIT group, only 16.7% exhibited moderate cognitive impairment. The results demonstrated a strong direct correlation between cognitive impairment as measured by the MMSE test and the levels of TSH, fT4, anti-TG and anti-TPO antibodies, 25-OH vitamin D, and brain-derived neurotrophic factor (BDNF). Furthermore, there was a strong direct correlation between cognitive impairment and fT4, and a weak direct correlation between 25-OH vitamin D levels. Furthermore, an inverse correlation was identified between the MMSE test and blood TSH levels [24]. George KM et al. also conducted a study to investigate the association between thyroid dysfunction, particularly autoimmune thyroid disease (AIT), and the risk of developing dementia. Their study showed that AIT itself was not significantly associated with an increased risk of dementia, but the study showed that a spectrum of thyroid dysfunction was associated with a different risk of dementia. Subclinical hypothyroidism was associated with a reduced risk of dementia. In contrast, overt hyperthyroidism was associated with an increased risk of dementia, compared with people with normal thyroid function (euthyroidism) [21]. Association patterns between hypothyroidism, hyperthyroidism, and cognitive impairment are illustrated in Figure 2 and Figure 3, indicating possible links rather than definitive causal effects. The findings suggest a potential association between thyroid disease and cognitive function, which may highlight the relevance of monitoring thyroid health as part of cognitive care. These associations underscore the importance of further research into thyroid disease management as part of dementia risk mitigation strategies, though direct evidence of treatment efficacy is not yet established.

### 3.2. Evidence of No Link Between Thyroid Disease and Cognitive Impairment

However, not all studies have reached specific conclusions on the effects of thyroid dysfunction on cognitive impairment and dementia. Kaur H et al. found that older patients with subclinical hypothyroidism did not differ in cognitive function from those without subclinical hypothyroidism, but the study sample was small, and the diagnosis of subclinical hypothyroidism was not well defined [23]. Wang Y et al. performed a comprehensive analysis to identify potential risk factors for dementia, initially including thyroid diseases (hypothyroidism and hyperthyroidism) among other chronic diseases that may affect cognitive function, such as diabetes, mental illness, and hearing loss. After adjusting for demographic and social variables, thyroid disease was not found to significantly increase the risk of dementia when combined with several other factors, suggesting that its role may not be direct or may be masked by stronger risk factors [25]. In the study by Van Vliet NA et al. involving 74,565 individuals, the vast majority, 66,567 (89.3%), had normal thyroid function. The study found no significant association between thyroid dysfunction and cognitive performance, including executive function, memory, or dementia risk [19]. These conflicting results highlight the complexity of the etiology of dementia and suggest that thyroid disease, broadly defined, is not an independent determinant of dementia risk, but type and duration of dysfunction should be considered.

## 4. Discussion

Despite extensive research, the link between thyroid function and cognition remains unclear. Studies show thyroid disorders cause cognitive impairment, with proposed mechanisms including Tau hyperphosphorylation, neuronal reduction, DNA hypermethylation, hippocampal shrinkage and neuron death, altered brain bioelectricity, decreased Na-K-ATPase activity, impaired brain metabolism and glucose uptake, altered neural activity, increased Aβ levels, inflammation, oxidative stress, cell death, altered gene expression, changes in synaptic plasticity, and reduced cerebral blood flow [1]. Thyroid hormones, particularly T4, play a critical role in neurodevelopment and cognitive function maintenance by influencing gene expression related to brain development (e.g., APP and BDNF genes). These hormones regulate synaptic plasticity, myelination, and neurotransmitter activity, which are essential for memory and executive function. Dysregulation in T4 levels may lead to impaired glucose metabolism and oxidative stress, thereby affecting cognitive health [1]. Given the complex effects of thyroid function on cognition and possible reverse causality, long-term prospective cohort studies are crucial, as they can clarify the relationship between thyroid dysfunction and cognitive decline, considering variables like age, sex, comorbidities, and medication use [27]. Reverse causality may contribute to the observed association between thyroid dysfunction and cognitive decline. For instance, cognitive impairment may affect hormonal regulation, thereby altering thyroid function, or early thyroid dysfunction symptoms might initially present as cognitive deficits. Chen Z et al. found a strong link between thyroid disease and cognitive impairment in subcortical ischemic vascular disease (SIVD). As cognitive function declined, serum TT3 and free triiodothyronine levels decreased, while serum TSH levels increased, correlating positively and negatively with MMSE scores, respectively. They concluded that thyroid function and hormone levels are risk factors and biomarkers for cognitive impairment in SIVD [28]. A 2017 study by Ma M et al. assessed the link between fT4, TSH, and anti-thyroid antibody levels and cognitive function in 383 postmenopausal women aged 50–65. Most had normal TSH and T4 levels, but the average neurocognitive scores were below normal, particularly in executive function, data processing speed, and complex attention. Over 50% scored below the norm in multiple cognitive domains. Cognitive scores decreased by 0.30 points per 0.1 mU/L increase in TSH and by 0.18 points per 1 nmol/L increase in TT4. There was also a significant negative correlation between anti-thyroid antibodies and cognitive function [29]. A 2023 meta-analysis by Ma LY et al. showed that hyperthyroidism (HR 1.14, 95% CI = 1.09–1.19) and subclinical hyperthyroidism (HR 1.56, 95% CI = 1.26–1.93) are associated with an increased risk of dementia. However, hypothyroidism (HR 0.93, 95% CI = 0.80–1.08) and subclinical hypothyroidism (HR 0.84, 95% CI = 0.70–1.01) were not linked to dementia risk. This review aligns with some previous studies, such as Pasqualetti et al., which reported a significant increased risk of cognitive impairment with subclinical hypothyroidism, while other studies by Akintola et al. and Tang et al. did not find this association [30,31,32]. A 2023 meta-analysis by Li-Yun et al. found that hyperthyroidism and subclinical hyperthyroidism may increase the risk of dementia, while hypothyroidism and subclinical hypothyroidism do not [33]. Recent guidelines emphasize the importance of thyroid function monitoring as part of comprehensive geriatric assessments, especially for patients with cardiovascular risk factors, given the potential impact on cognitive health [2,28]. The WHO’s 2019 guidelines on reducing cognitive decline emphasize comprehensive monitoring of modifiable risk factors, including thyroid function. The guidelines recommend integrating thyroid assessments in geriatric and cardiovascular risk evaluations, as unaddressed thyroid dysfunction can contribute to cognitive impairment and accelerate dementia onset. These evaluations support early intervention strategies in high-risk groups, particularly elderly individuals with comorbidities such as cardiovascular disease, aligning with dementia prevention efforts in aging populations. These guidelines underscore the growing focus on personalized, preventative healthcare as dementia cases continue to rise globally. The results observed in the literature remain controversial. This may be due to different samples, a very heterogeneous distribution of patients, and different units of measurement. However, this does not negate the fact that thyroid patients should also be screened for cognitive impairment.

## 5. Conclusions

The review reveals a complex and ambiguous relationship between thyroid dysfunction and cognitive impairment. While some studies link thyroid disorders, particularly hypothyroidism, to an increased dementia risk, others find inconsistent associations or suggest that the relationship varies by thyroid disorder type or cognitive domain. Variability in findings may stem from differences in study populations, cognitive assessment methods, or thyroid disorder types. Given the link between thyroid dysfunction and cognitive impairment, routine screening for thyroid function in patients presenting with cognitive symptoms is recommended, particularly for older adults. Current guidelines suggest annual screening for TSH levels in cognitively impaired patients to allow timely intervention. Nevertheless, cognitive assessments for thyroid patients could include screening intervals aligned with thyroid monitoring schedules, using tests such as the Mini–Mental State Examination (MMSE) to detect early cognitive changes. Further research is needed to elucidate the mechanisms underlying these associations and to determine whether thyroid dysfunction may be a modifiable risk factor for dementia.

## Figures and Tables

**Table 1:** Keyword combinations and search in databases.

Keyword Combinations Used	Search in the Following Databases (n)		
	PubMed	Cochrane	ScienceDirect
“thyroiditis” and “cognitive decline”	1	5	20
“thyroiditis” and “cognitive dysfunction”	4	11	20
“thyroiditis” and “cognitive impairment”	3	16	63
“thyroiditis” and “dementia”	1	12	53
“thyroid disease” and “cognitive decline”	3	2	84
“thyroid disease” and “cognitive dysfunction”	2	2	58
“thyroid disease” and “cognitive impairment”	6	4	217
“thyroid disease” and “dementia”	5	5	262
“hypothyroidism” and “cognitive decline”	4	1	163
“hypothyroidism” and “cognitive dysfunction”	19	2	134
“hypothyroidism” and “cognitive impairment”	18	1	141
“hypothyroidism” and “dementia”	19	7	237
“hyperthyroidism” and “cognitive decline”	3	1	14
“hyperthyroidism” and “cognitive dysfunction”	5	0	49
“hyperthyroidism” and “cognitive impairment”	8	1	147
“hyperthyroidism” and “dementia”	5	0	220

**Table 2:** Quality assessment of the articles using the Newcastle–Ottawa Quality assessment scale.

Order No.	Study Authors, Year	Selection			Comparability	Outcomes
		1	2	3	4	5
1.	Kim JH et al., 2022 [18]	*	*	*	**	*
2.	van Vliet NA et al., 2021 [19]	*	*	*	**	*
3.	Thvilum M et al., 2021 [20]	*	*	*	**	*
4.	George KM et al., 2019 [21]	*	*	*	**	*
5.	Sipilä PN et al., 2020 [22]	*	*	*	**	*
6.	Kaur H et al., 2021 [23]	*	*	*	**	*
7.	Kamyshna II et al., 2022 [24]	*	*	*	*	*
8.	Wang Y et al., 2023 [25]	*	*	*	**	*
9.	Folkestad L et al., 2019 [26]	*	*	*	**	*

**Table 3:** Main characteristics of the studies.

	Authors, Year	Country	Target Group	Sample Volume	Thyroid Diseases Assessed	Study Results
Cohort studies						
1.	van Vliet NA et al., 2021 [19]	Belgium, Germany, Netherlands, France, UK, USA, Mexico, Korea	Older people living in the community	74,565	Hypothyroidism, hyperthyroidism, subclinical hypothyroidism, and subclinical hyperthyroidism	The study found no significant association between thyroid dysfunction and cognitive performance, including executive function, memory, or dementia risk.
2.	Thvilum M et al., 2021 [20]	Denmark	Hypothyroid adults	791,669	Hypothyroidism	Hypothyroidism had an elevated risk of dementia (OR 1.22 and 95% CI: 1.17–1.27) DNPR cohort, but after accounting for pre-existing comorbidities, an adjusted HR was 0.82 (95% CI: 0.79–0.86). The risk was significantly elevated in individuals younger than 56 years (OR 2.03, 95% CI: 1.62–2.53), whereas it was not statistically significant in those aged 56 or older. An elevated TSH level at six months was linked to a 1.12 SD increase in the likelihood of developing cognitive impairment (95% CI: 1.07–1.16).
3.	George KM et al., 2019 [21]	USA	Adults living in the community	12,481	Hypothyroidism, hyperthyroidism, subclinical hypothyroidism, and subclinical hyperthyroidism	Subclinical hypothyroidism was associated with a reduced risk of dementia (HR 0.74; 95% CI: 0.60 to 0.92). In contrast, overt hyperthyroidism was associated with an increased risk of dementia, (HR 1.40; 95% CI: 1.02–1.92).
4.	Sipilä PN et al., 2020 [22]	Finland, UK	Adults living in the community	283,414	Hypothyroidism	Hypothyroidism nearly doubled dementia risk (HR 1.94; 95% CI: 1.59–2.38).
5.	Kamyshna II et al., 2022 [24]	Ukraine	Women with hypothyroidism or autoimmune thyroiditis	153	Hypothyroidism and autoimmune thyroiditis	The results demonstrated a strong direct correlation between cognitive impairment as measured by the MMSE test and the levels of TSH, fT4, anti-TG and anti-TPO antibodies, 25-OH vitamin D and brain-derived neurotrophic factor (BDNF) (*p* < 0.001). Furthermore, there was a strong direct correlation between cognitive impairment and fT4 (*p* < 0.001), and a weak direct correlation between 25-OH vitamin D levels (*p* < 0.001). Furthermore, an inverse correlation was identified between the MMSE test and blood TSH levels (*p* < 0.001).
6.	Folkestad L et al., 2019 [26]	Finland, UK	Hyperthyroid patients	291,725	Hyperthyroidism	While having Graves’ disease and toxic nodular goiter, for every six months of TSH reduction, there was a notable increase in the risk of all-cause dementia (HR 1.16; 95; CI: 1.12–1.22).
Case control studies						
1.	Kim JH et al., 2022 [18]	Korea	Alzheimer’s patients	82,365	Hypothyroidism, hyperthyroidism, thyroiditis	Higher prevalence of thyroid disorders in individuals with Alzheimer’s disease (AD): hypothyroidism (OR) = 1.14, 95% CI = 1.00–1.30), thyroiditis (OR = 1.22, 95% CI = 1.05–1.40), hyperthyroidism (OR = 1.13, 95% CI = 1.01–1.28).
2.	Kaur H et al., 2021 [23]	India	Persons >60 years	200	Subclinical hypothyroidism	Older patients with subclinical hypothyroidism did not differ in cognitive function from those without subclinical hypothyroidism.
Cross-sectional study						
1.	Wang Y et al., 2023 [25]	China	Persons >60 years	2595	Hyperthyroidism, hypothyroidism	Thyroid disease was not found to significantly increase the risk of dementia when combined with several other factors (*p* = 0.313).

## Appendix A

**Table A1:** Newcastle–Ottawa quality assessment scale.

Part of the Criteria		Criteria	Description of the Evaluation
Selection	1	Were the criteria used to include patients described?	1 “⁎”—the criteria are given
	2	Was the sample representative?	1 “⁎”—the selected patients were representative of the population. 0 “⁎”—patients were selected without justification, or the choice of patient group is not described.
	3	How was the comparison group selected?	1 “⁎”—the selected patients were demographically matched to the subjects. 0 “⁎”—patients selected are not from the same population as the subjects, or the choice of patient group is not described.
Comparability	4	Comparability between groups assessed by study design or group difference analysis	2 “⁎”—no differences between groups or controlled differences (based on patient characteristics: age, gender). 1 “⁎” if 1 of the 2 patient characteristics was not reported (even if there are no more differences between the groups and the others are controlled). 0 “⁎”—different groups of subjects.
Outcomes	5	Graduation assessment	1 “⁎”—information provided. 0 “⁎”—no information provided.
